# A High-Performance Gamma Spectrometer for Unmanned Systems Based on Off-the-Shelf Components

**DOI:** 10.3390/s22031078

**Published:** 2022-01-29

**Authors:** Andrea Chierici, Andrea Malizia, Daniele Di Giovanni, Riccardo Ciolini, Francesco d’Errico

**Affiliations:** 1 Department of Industrial and Civil Engineering, University of Pisa, 56122 Pisa, Italy; riccardo.ciolini@unipi.it (R.C.); francesco.derrico@unipi.it (F.d.); 2 Department of Industrial Engineering, University of Rome Tor Vergata, 00133 Rome, Italy; daniele.di.giovanni@uniroma2.it; 3 Department of Biomedicine and Prevention, University of Rome Tor Vergata, 00133 Rome, Italy; 4 School of Medicine and Surgery, Unicamillus-Saint Camillus International University of Health Sciences, Via di S. Alessandro, 8, 00131 Rome, Italy; 5 Yale Center for Emergency Preparedness and Disaster Response, New Haven, CT 06519, USA

**Keywords:** UAV, radiation detection, gamma spectroscopy, emergency, first responders

## Abstract

Since the Fukushima Daiichi Nuclear Power Plant accident in March 2011, the technology available for unmanned aerial vehicles (UAVs) for radiation monitoring has improved greatly. Remote access to radiation-contaminated areas not only eliminates unnecessary exposure of civilians or military personnel, but also allows workers to explore inaccessible places. Hazardous levels of radioactive contamination can be expected as a result of accidents in the nuclear power industry or as a result of the intentional release of radioactive materials for terrorist purposes (dirty bombs, building contamination, etc.). The possibility to detect, identify, and characterize radiation and nuclear material using mobile and remote sensing platforms is a common requirement in the radiation sensing community. The technology has applications in homeland security and law enforcement, customs and border protection, nuclear power plant safety and security, nuclear waste monitoring, environmental recovery, and the military. In this work, the authors have developed, implemented, and characterized a gamma-ray detection and spectroscopy system capable of operating on a UAV. The system was mainly developed using open-source software and affordable hardware components to reduce development and maintenance costs and provide satisfactory performance as a detection instrument. The designed platform can be used to perform mapping or localization tasks to improve the risk assessment process for first responders during the management of radiological and nuclear incidents. First, the design process of the system is described; the result of the characterization of the platform is then presented together with the use of the prototype installed on a UAV in an exercise simulating a radiological and nuclear contamination scenario.

## 1. Introduction

### 1.1. Motivation

Unmanned aerial vehicles (UAVs) for chemical, biological, radiological, and nuclear (CBRN) agent monitoring has been successfully used in recent times, mainly thanks to the advancement in the field of robotics and miniaturization of detection technologies [1,2,3].

In the context of radiation monitoring, the use of UAVs has greatly increased after the incident at the Fukushima Daiichi Nuclear Power Plant (FDNPP) in March 2011, where radiation mapping and monitoring tasks were necessary to localize and identify the contaminating agents and areas [4,5,6,7,8]. Indeed, reducing the amount of time for detection operations in the hot zone through remote access to a radiation-contaminated area not only precludes unnecessary exposure of civil or military personnel, but it also allows the monitoring of unreachable areas. One of the main consequences of this approach is the improved capability to collect signals by placing the sensors closer to certain locations of interest, increasing the detection efficiency; approaching a location of interest from different directions (to reduce the attenuation by shielding materials); and hovering in a location for much longer than a human operator could safely or comfortably do.

This technology has relevance to applications in homeland security and law enforcement, customs, and border protection, nuclear power plant safety and security, nuclear waste monitoring, environmental recovery, and the military, as human operators using handheld devices may not be able to safely access the locations involved in such activities [9,10,11,12,13,14].

There are different drone technologies available, which can be grouped into two main categories: fixed-wing systems (which can reach very high speed but suffer from poor spatial resolution when conducting the surveys) and rotorcraft (single or multi-engine systems that can move slowly or hover in a stationary position). Rotorcrafts are suitable for carrying out radiation monitoring tasks in small and confined areas and where manoeuvrability is mandatory [5,15].

The development of a UAV-based remote detection system includes several aspects ranging from cost, size, availability, complexity, and power consumption, which may affect the overall efficacy of the specific radiation detection technique. The key aspects that determine the signal-to-noise ratio (SNR) are the measurement time, the distance between the source and the detector, and the attenuation of the materials in between. 

The UAV-based approach is quite different from any other aerial vehicle detection approach because of several aspects. There are a few limits, such as the limited detector size and weight that can be employed. For example, large NaI(Tl) crystals, as well as most cooled HPGe detectors, are too big to be carried by a typical multirotor UAV. However, collection efficiency can be increased by inspecting the source from a very short distance. UAVs can fly at very low altitudes and speeds (improving SNR and the spatial resolution of the inspection); moreover, they have relatively low costs compared to manned systems, and they are easy to manoeuvre and operate in confined spaces.

### 1.2. Radiological and Nuclear Threats

To remotely sense the presence of radioactive and nuclear (RN) materials, a knowledge of the background activity, the radioactive signatures exhibited by the materials of interest, and other potential noise sources is required.

The most significant indicators for the presence of radiation contamination are gamma-ray and neutron emissions associated with hazardous materials. Hazardous materials include fissile isotopes that may be used to make nuclear weapons, or highly active industrial sources that could be employed in a radiological dispersal device (RDD), radiological exposure device (RED), or improvised nuclear device (IND) [16,17,18].

Although several novel and potential hazardous materials may be found nowadays, uranium and plutonium [19,20] are still the main actors in the context of nuclear safety and security because they are the most common materials used to fabricate nuclear weapons and produce nuclear fuel. The most measurable emissions of these elements are the gamma rays associated with the radioactive decay of the parent and daughter isotopes, the neutrons released during spontaneous fission, the gamma rays resulting from the capture reaction of thermal neutrons in nearby materials, and impurities found naturally in a material or caused by a specific processing. Active interrogation through exposure to high energy X-ray and neutron beams can also be employed to trigger a more significant radioactive signature compared to spontaneous emissions [21,22,23].

The specific activity, half-life, decay, chemical form, and availability make certain isotopes much more interesting as threat source material for a RDD, RED, or IND. Recommendations on the radionuclides of interest, as well as the levels of activity that may trigger security measures, have been made by the IAEA (International Atomic Energy Agency) and the U.S. Nuclear Regulatory Commission [24]. Specifically, the activity level for many radionuclides to be considered hazardous materials is between 37 and 370 GBq. Although such activity values may seem large, isotopes such as ^137^Cs and ^60^Co have specific activities on the order of TBq∙g^−1^, thereby requiring just milligrams of material to constitute a threat [25]. Moreover, such radionuclides are widespread because of their large use in the industrial and medical sectors.

In this work, an RN detection system was realized and characterized by the authors using mostly open-source software, off-the-shelf components, and custom-made electronics. The system is lightweight, compact, inexpensive, and battery-powered. It was designed with the goal of being used in a remote sensing and mobile platform, and specifically to be mounted on rotorcraft UAVs of the mini category. The platform was provided with different tools, such as a radio frequency (RF) trans receiver, a memory card support, a temperature sensor, and the electronics required to manage the signals generated following the interaction of the detectors with ionizing radiation. Multiple detection channels operating at the same time can also be added to the setup, potentially increasing the statistical confidence of the detection, and potentially improving the effectiveness of the source localization and identification tasks.

## 2. Materials and Methods

In this work the focus was set on the detection of gamma rays because of their highly penetrating nature; although neutrons share a similar behaviour, they have not been considered in this application, but their detection could be considered in future activities. Alpha and beta particles (and in more general charged particles) have been discarded because of their short-range in air.

Although there are different radiation detection technologies available, scintillation detectors are the ones that offer high detection efficiency in a small active volume with a reduced economic effort [26,27]. Following the interaction with ionizing radiation, scintillators emit visible or ultraviolet light pulses. Such pulses can be converted into electrical signals by low-level light detectors, such as photodiodes, photomultiplier tubes, avalanche photodiodes, or silicon photomultipliers (SiPMs). By collecting the histogram of the light pulses, the energy spectrum of a gamma-ray field can be measured in a process called scintillation spectroscopy.

**Figure 1:**
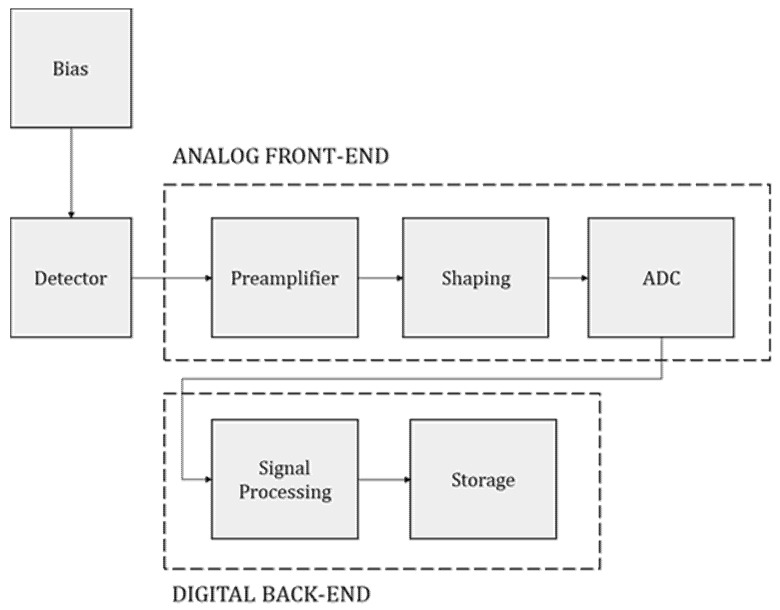
General scheme of a PDFE.

To gather and convert such signals, several approaches can be employed depending on the available hardware. In our application a peak detector (PKD) was used to temporarily store the amplitude of the voltage signals generated by the light pulses; the signals were then digitized by means of an analog-to-digital converter integrated into a low-cost microcontroller acting as a data acquisition unit. Before the PKD, some core components are commonly found, such as a preamplifier-shaper-discriminator chain that we implemented in a single custom-made printed circuit board (PCB). The system used to acquire, convert, and store the nuclear signals is called particle detection front end (PDFE), shown in Figure 1.

### 2.1. Scintillation Detector and Voltage Bias

The choices regarding the scintillator material, the SiPM, and the SiPM bias module are strongly interdependent. We employed a 10 mm × 10 mm × 40 mm CsI(Tl) scintillator crystal procured from Epic Crystal [28]. The crystal was wrapped with four layers of BC-642 PTFE Reflector Tape from Saint-Gobain and optically coupled to a SiPM using BC-630 silicon optical grease [29]. It is important to ensure that the PTFE does not touch the silicone grease, which may result in a worsened performance of the PTFE as a reflector.

Caesium iodide (CsI) was selected as a detector material because of its high gamma-ray absorption coefficient thanks to its relative high density and atomic number. Moreover, it is quite rugged and well-suited for applications and environments where severe shock conditions are encountered (for example, on a UAV). When used in scintillation spectroscopy, CsI is commonly doped with sodium or thallium. Compared to NaI, CsI is only slightly hygroscopic. CsI(Tl) is also one of the brightest scintillators, featuring a maximum of the broad emission roughly at 550 nm; the emission is, therefore, well-suited for photodiode or SiPM readout [27].

The dimensions of the crystal were chosen to provide good absorption coefficient for a 662 keV ^137^Cs gamma ray directly incident on the long axis of the crystal, or to maximize the counting effectiveness of the detection system, depending on the positioning of the crystal. However, the ratio of the counts in the photopeak to the total number of counts (peak-to-total ratio) is a function of the geometry of the detector itself.

When the crystal width is small compared to the mean free path of secondary scattered particles, then these secondaries can escape without contributing to the photopeak [26]. The result is a low peak-to-total ratio that varies strongly based on the relative orientation of the crystal and source. As the SiPM has a smaller active area compared to the exit window of the crystal, a small chamfer was realized at the end of the crystal to improve the light collection efficiency.

We employed a 6 mm × 6 mm s13360-6050PE SiPM from Hamamatsu, which was attached to a printed circuit board (PCB). The SiPM high-voltage (HV) bias was supplied by a positive output programmable high voltage power supply.

### 2.2. Analog Readout

**Figure 2:**
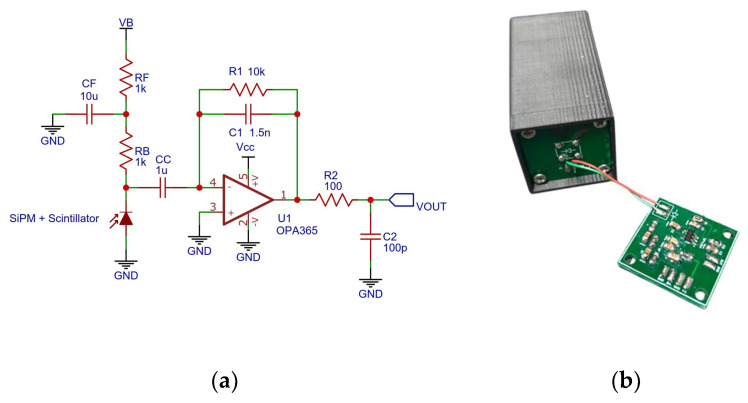
CSA schematic (**a**) and realization (**b**).

A dedicated PCB was designed for the charge sensitive amplifier (CSA) with a specified pulse height sensitivity of 0.67 mV∙pC^−1^ and a decay time of 15 μs (Figure 2). The board is powered by a single cell 3.7 V LiPo battery, greatly reducing the complexity of the circuit. The operational amplifier chosen (OPA365, from TI), was selected because of its high slew rate, relatively large bandwidth, rail to rail input-output characteristics (less than 10 mV from the rails), and negligible input bias currents.

To keep the number of components to a minimum (saving space, cost, and power consumption), the single stage CSA was directly interfaced to the multi-channel analyser (MCA), which is described in the next subsection. Indeed, preamplifiers are often followed by shaping amplifiers to increase the SNR and to modify the timing characteristics of the incoming pulse; when shaping amplifiers are employed, additional circuitry is also added, such as baseline restoration circuits. However, in our application, the intrinsic and high gain of the SiPM was considered enough to achieve an adequate SNR already from the output of the CSA. Moreover, since the size of the scintillator used is small, the pile-up probability of the pulses is also low.

### 2.3. Multi-Channel Analyser Based on Peak Detection

**Figure 3:**
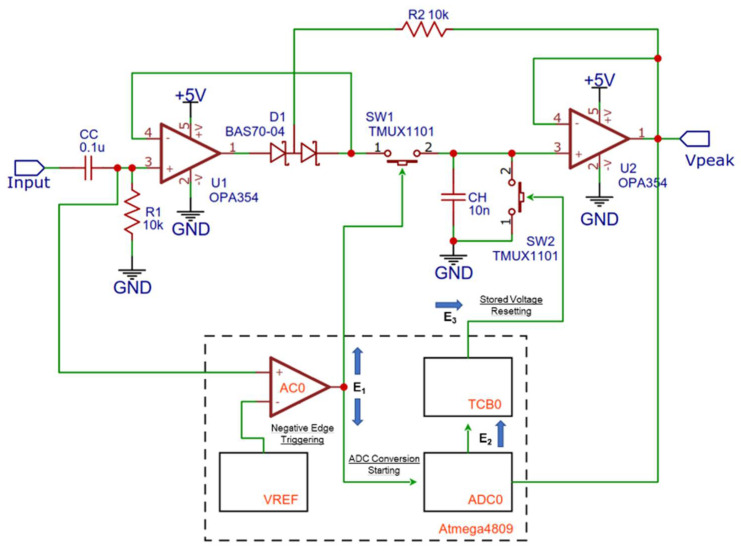
Architecture and functional scheme of the multi-channel analyser based on peak detection.

In our implementation, a MCA using a peak detection circuit built upon on a high-speed operational amplifier (OPA365/OPA354, from TI) [30,31] as a first stage input buffer was developed, as shown in Figure 3; the peak value is stored on a low leakage hold capacitor (CH) and can be reset through a single pole single throw switch (SW2, a TMUX1101 from TI) [32]. A second switch of the same model (SW1) is present on the input signal line of the circuit to grant a non paralysable behaviour to the acquisition system in case of very high counting rates. The logical operation of the circuit is managed by an Atmega4809 microcontroller (from Microchip) [33].

The input signal is compared to a voltage reference (scaled by the internal digital-to-analog converter) by means of the comparator peripheral integrated in the Atmega4809. When the input signal becomes lower than the threshold (negative edge triggering), an event (E1) is generated and routed through the event system peripheral (EVSYS) to the ADC peripheral (ADC0). Negative edge triggering of the comparator guarantees that the peak value of the pulse has been reached before starting a new conversion. Before starting the conversion, SW1 is also opened in order to reject incoming pulses when a conversion is already ongoing. When the ADC conversion (set to 10 bits resolution or 1024 channels by default) is completed, the new value is added to a temporary spectrum by the CPU. As shown later, the temporary spectrum is going to be requested by another microcontroller acting as a master every second; when the transfer is completed, then the temporary spectrum is reset. Upon completion of the ADC conversion, a new event is generated (E2) that triggers a 16 bit general purpose timer/counter peripheral (TCB0). The timer/counter, configured in single shot mode, acts as a monostable multi-vibrator generating a digital pulse (having an arbitrary duration T_p_ longer than the time required to reset the peak detector). The digital pulse triggers SW2 through a third event (E3) routed from the timer/counter to a digital output pin of the microcontroller. Because the EVSYS is used to interconnect the peripherals, the behaviour of the circuit is completely predictable, and the CPU is free to manage the update of the temporary spectrum and the communication with the master.

### 2.4. Master Unit, Other Modules, and Data Flow

In this subsection, the unit acting as the master of the system and the data flow toward and from the other modules present on the platform (the MCAs, the RF module, the HV power supply, the temperature sensor, and the SD card module) are described.

The unit acting as master is a microcontroller of the same model used for the implementation of the MCAs, an Atmega4809. The micro was programmed to communicate with the other modules through I^2^C and SPI serial communication protocols, to manage the spectral data, and to communicate with a receiving ground station when needed. By default, the master requests the temporary spectra collected by the MCAs every second, which are then packed in 32 B wide payloads and transmitted to ground through the RF communication module. As each MCA is able to collect the spectrum independently from the others, several RN detectors may be installed on the platform at the same time. The main limits when using several detectors are both physical and technological, such as: space, weight, power consumption, and management of the data flow toward the ground station. In the current implementation, where only two detectors were used, the number of channels was set to 1024; if several spectra must be collected and managed at the same time, the channel resolution may be lowered to 256 to limit the amount of data to be transferred through the RF communication channel. Because at this time a CsI(Tl) scintillation detector is being used, a channel resolution between 256 and 1024 was considered already adequate to perform basic gamma spectroscopy without losing the energy resolution information.

A shielded version of the RF trans receiver module (nRF24L01+, Nordic Semiconductor) [34] was used to reduce the effect of electro-magnetic interference on the communication. The module operates at 2.4 GHz, it features a maximum RF baud rate of 2 MSPs, and the maximum RF payload size for a single data packet is 32 B. It can reach a communication distance of up to 2 km. A similar module, acting as RF receiver, was employed to decode and transfer the received data to the ground station (for example, a generic laptop). Roughly 100 ms are required to collect and transmit a 1024 channel spectrum, therefore if the data coming from several RN detectors must be exchanged on the same bus, a refresh rate lower than 1 Hz may be required.

While managing the spectral data and the communication toward ground, the master also collects the temperature, pressure, and humidity information from the dedicated sensor (BME280, from Bosch) [35]. The sensor features the following dynamic ranges: between −40 °C and +85 °C for the temperature, between 0% and 100% RH for the humidity, and between 300 hPa and 1100 hPa for the pressure. In the current implementation, only the temperature information (precision equal to 0.01 °C and accuracy equal to ±1 °C) is actively used to compensate the gain (and the other characteristics) of the SiPM.

The low noise and low ripple HV power supply was designed using a boost converter integrated circuit (MAX5026, from Maxim Integrated) [36]. The power supply is controlled digitally through a digital-to-analog converter (MCP4725, from Microchip) [37] scaling a 5 V precision voltage reference (REF5050, from TI) [38], which allows us to set the HV output between 0 V and 65 V. Such voltage is needed to keep the SiPM in breakdown mode. The SiPM chosen features a nominal breakdown voltage equal to 53 ± 5 V @ T = 25 °C, and the suggested overvoltage is equal to 3 V. The correction factor reported by the manufacturer is 54 mV/°C, and if the temperature rises, then bias voltage must be increased proportionally to keep the gain, dark count, cross-talking, and photon detection efficiency features of the SiPM constant. The parameters reported by the manufacturer are used by the master to periodically compute the bias voltage needed by the SiPM over temperature.

A SD card module is also present on the platform, and if requested the master will store all the spectral, temperature, humidity, and pressure data locally at a speed of roughly 250 kB/s.

**Figure 4:**
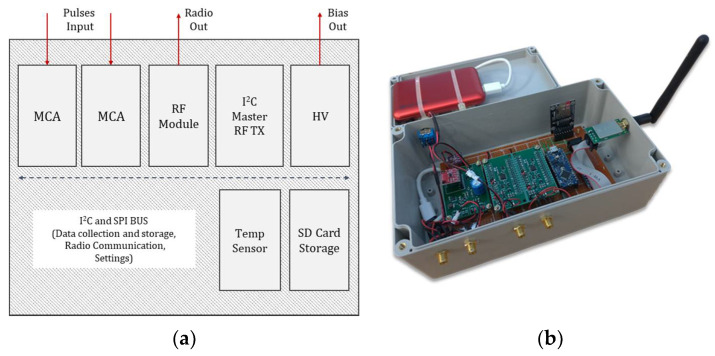
Architecture (**a**) and prototype (**b**) of the data acquisition and management system.

The prototype consisting of a data acquisition unit (DAQ) and all the described modules was designed, realized, tested, and applied in the field. The scheme and the actual prototype are shown in Figure 4.

**Figure 5:**
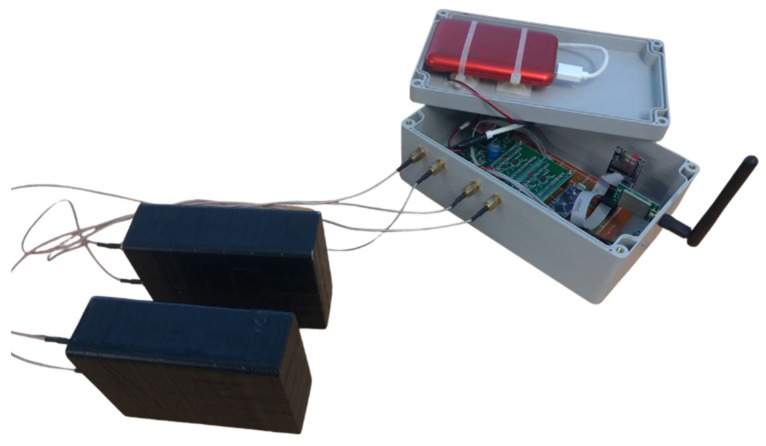
The detection system consisting of two detection modules connected to the data acquisition and management module.

The whole system consists of two detection modules and the DAQ/RF unit has a weight of less than 1 kg and power consumption of less than 300 mA (Figure 5 and Table 1). The system is powered by an external LiPo battery having a capacity of 10 Ah. UAVs often have flight autonomy on the order of tens of minutes, whereas our detection and data collection/processing/transmission system can operate for more than 10 h continuously before recharging. Some custom PCBs have been designed and realized to develop the prototype, and the reported cost only considers the printing process of the boards used.

### 2.5. Graphical User Interface

**Figure 6:**
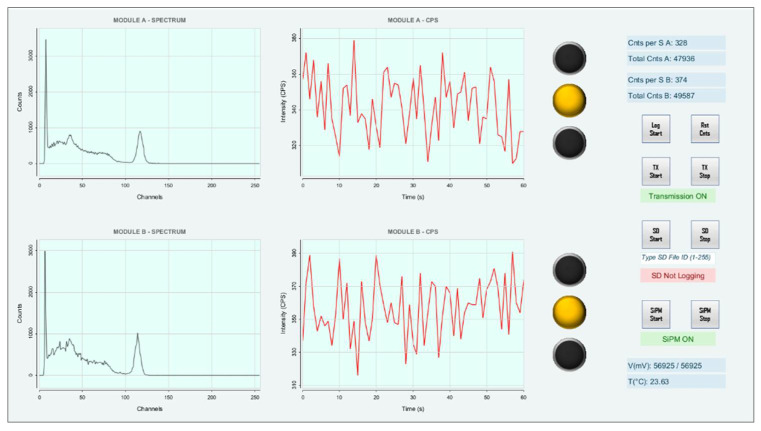
Graphical user interface.

A basic graphical user interface (GUI) was designed to plot the RN spectra and counts per second (CPS) received by the RF receiving module (Figure 6). The GUI does not yet allow us to modify the parameters of the MCA, such as the gain or the calibration factors; it only allows us to check and save to specific log files the shapes of the received spectra and the trends of the CPS over time. A reset button was also implemented to reset the spectra when required. The goal is to add the required features to the next version of the GUI.

## 3. Results and Discussion

To investigate the behaviour of the analog front end and of the data acquisition system, the preamplifier was enclosed in an aluminium shielded box (1.6 mm thick wall) to minimize the potential effect of electromagnetic interference (EMI). Then, a lab-made sawtooth waveform generator (built on a SAMD21G18 Microcontroller) [39] was used to study our system’s linearity, calibration factors, and electronic noise. Known charges were injected into a testing capacitor coupled to the input of the CSA according to a wave frequency of 250 Hz. As the nominal value of the coupling capacitance was 3.3 nF (±5%), the values of the injected charge were given by:

(1) $$Q=V_{step}\times C_{test}$$

where $V_{step}$ is the amplitude of the sawtooth waveform (adjustable by means of the digital-to-analog converter found in the micro), and $C_{test}$ is the actual value of the coupling capacitance. After the analog front end, the data acquisition system was placed inside a separate box, and the signal generated by the CSA was fed into the MCA using SMA cables and connectors. By choosing the sawtooth amplitude accordingly, the charges injected were nominally set to 280 pC, 360 pC, 432 pC, 540 pC, 720 pC, 900 pC, 1.08 nC, 1.44 nC, 1.80 nC, 2.16 nC, 2.88 nC, and 3.24 nC. 

To check the shape of the output signal from the CSA and the noise level, the line was also split and read out by a LeCroy WaveAce 101 digital oscilloscope. To avoid unwanted counts triggered from pure electronic noise, a voltage threshold for the discriminators of 50 mV, corresponding to roughly 30 keV after energy calibration, was used.

### 3.1. Linearity and Electronic Noise Measurements

**Figure 7:**
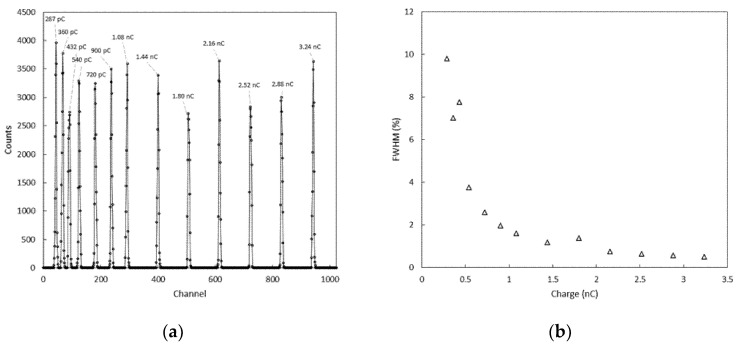
Histogram of the counts used to for the noise test (**a**); charge resolution (**b**).

**Figure 8:**
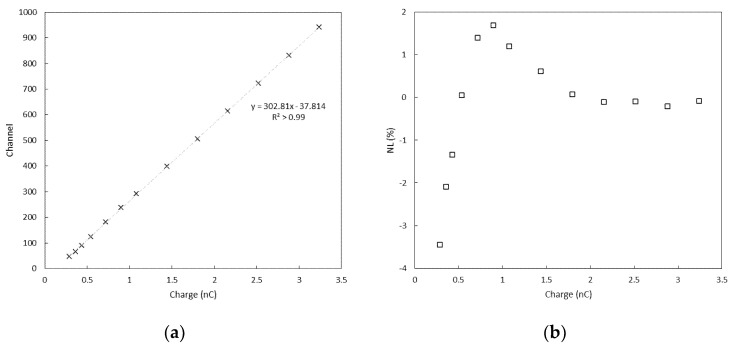
Results of charge calibration using a linear fit (**a**); relative residuals of the linear fit. The system shows less than 4% non-linearity through the dynamic range (**b**).

Results of the charge calibration of the CSA and MCA systems are shown in Figure 7 and Figure 8. The full width at half maximum (FWHM) of the distribution of the pulses can be used to estimate the noise associated with the conversion process. A FWHM of two channels, corresponding to about 5 mV, was found for all the test charges across the dynamic range, and a charge resolution (expressed as % FWHM) going from roughly 10% to less than 0.5% was estimated. Such values of charge resolution were considered adequate when the system is used to perform scintillation spectroscopy with a CsI(Tl) scintillation crystal, as a higher level of uncertainty can be expected from the processes of generation and collection of signal carriers [40,41]. The peak values of the distribution were also linearly fitted to the charges injected in the CSA, and the relative residuals to each fit are shown in Figure 8. The linear fit was appropriate for the present work; however, the response appears slightly non-linear toward low charge values. The non-linearity (NL), is defined as:

(2) $$\mathrm{NL}=100\;\cdot \;\frac{Q_{meas}-Q_{fit}}{Q_{meas}}$$

is better than 4% across the dynamic range of the test charges.

We conclude that the system can be used to acquire the signals produced by the CSA, and it is a promising solution to perform basic gamma detection and spectroscopy tasks.

### 3.2. Energy Calibration

The response of the detection system was tested by exposing the scintillators to ^137^Cs, ^60^Co, and ^241^Am sealed radioactive sources at the Laboratory of Nuclear Measurements, Department of Industrial and Civil Engineering, Pisa (Italy). From the spectra collected during the tests, it was possible to assess the presence of the typical spectral features of gamma scintillation spectroscopy, such as gamma photo-peaks, Compton valley, edge, continuum, backscatter, etc. The same data were then used to perform an energy calibration of the detection system.

**Figure 9:**
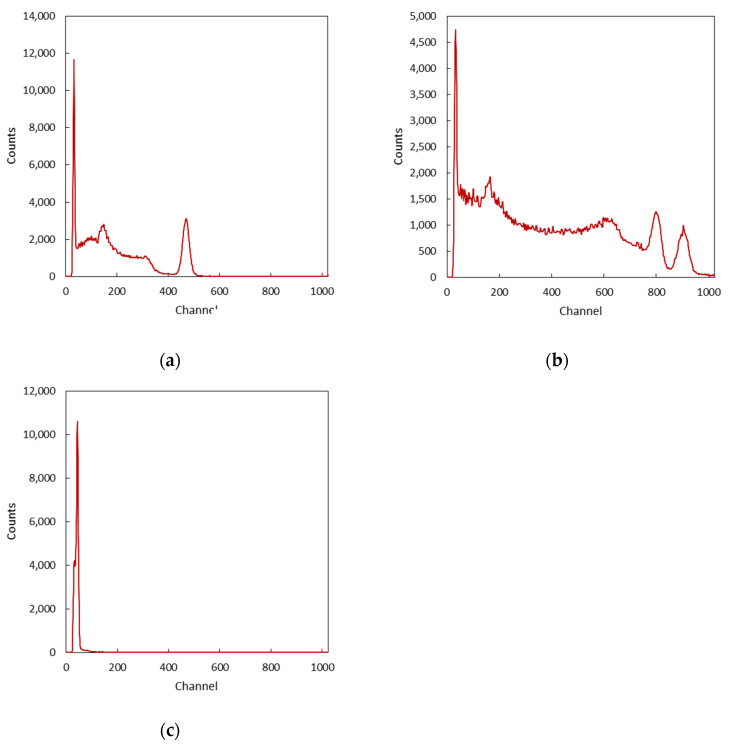
Measured spectra used for energy calibration: ^137^Cs (**a**), ^60^Co (**b**), ^241^Am (**c**).

**Figure 10:**
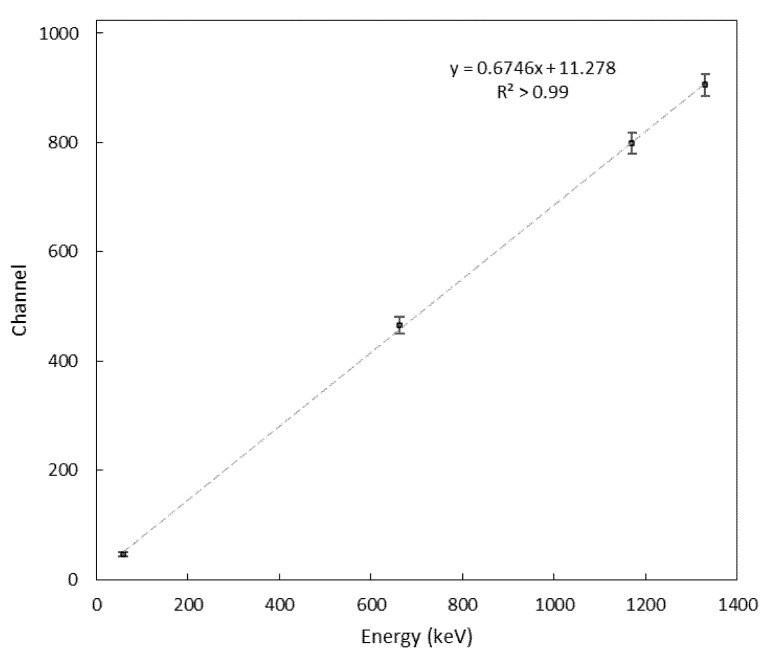
Energy calibration results, showing satisfactory linearity for the SiPM readout of the CsI(Tl) crystal. Non-linearity at lower energies may result from low energy non-linearity observed during charge calibration, but it is also consistent with expectations for CsI(Tl).

As shown in Figure 9, the gamma photopeaks for ^137^Cs (662 keV), ^60^Co (1.173 MeV and 1.332 MeV), and ^241^Am (59 keV) are clearly visible. The typical features of the spectra, such as the Compton valleys, edges, continuum, and backscatter are present as well. Moreover, the readout of the SiPM coupled to the CsI(Tl) crystal resulted sufficiently linear across the range of energies tested. A certain level of non-linearity at lower energies may result from low energy non-linearity observed during charge calibration, but it is also consistent with the behavior of CsI(Tl) (Figure 10 and Table 2) [30,31].

### 3.3. Photopeak Efficiency

**Figure 11:**
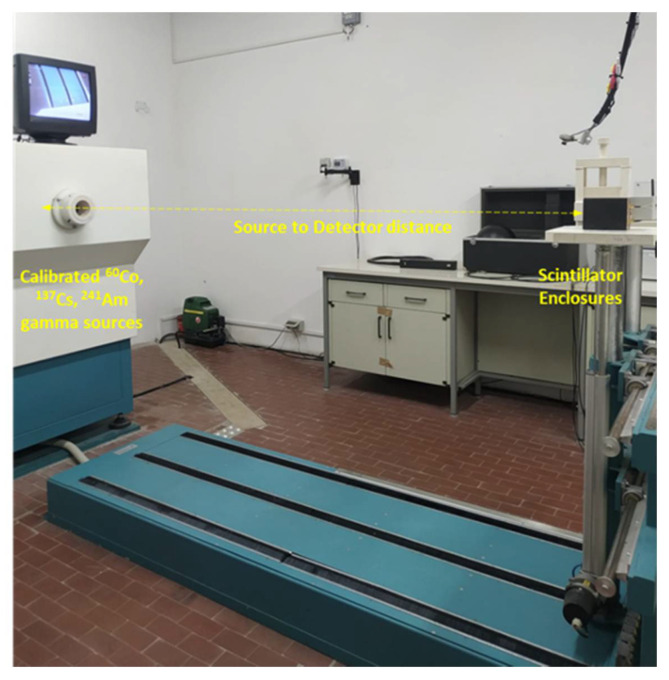
Setup of the measurements performed to investigate the energy dependence of the intrinsic photopeak efficiency of the detectors.

By analyzing the acquired spectra, the contribution of the photopeak area compared to the area associated with the Compton continuum can be assessed. Because we were using relatively thin crystals, different peak-to-total ratios of the areas are observed when a source is placed above the long axis of the crystal or next to its side. Source detector orientation also modifies the photopeak efficiency. The intrinsic photopeak efficiency is defined as the ratio between the number of recorded photopeak counts and the number of radiation quanta incident on the detector surface. In our application, the scintillator inside the enclosures were positioned to maximize the counting effectiveness, therefore the photopeak efficiency was investigated by exposing the 10 mm × 40 mm window. A future study may be carried out to find out the optimum size and positioning of the detector based on the specific application. The energy dependence of the intrinsic efficiency of the photopeak was investigated at CISAM (Joint Centre for Military Studies and Applications), San Piero a Grado (Italy), where calibrated ^137^Cs, ^60^Co, and ^241^Am gamma emitters were used to expose the detection system (both channels, namely A and B, were connected and activated), as shown in Figure 11 and Table 3.

**Figure 12:**
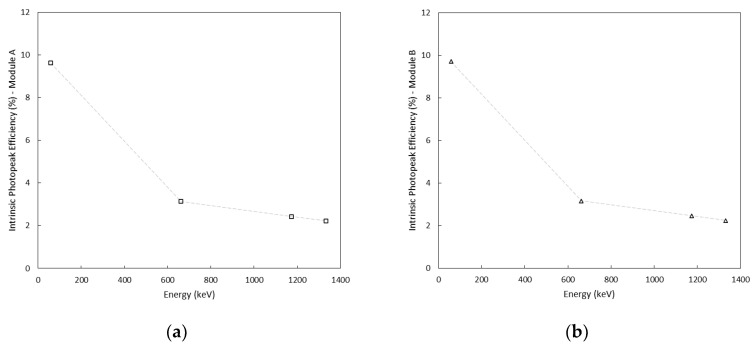
Intrinsic photopeak efficiency at different energies for module A (**a**) and module B (**b**).

The resulting photopeak efficiencies for both modules are presented in Figure 12.

### 3.4. In Flight Testing

The behaviour of the radiation detection system mounted under a UAV—a DJI Matrice 200—was first tested through measurements conducted at the Joint NBC Defence School in Rieti (Italy), which provided a 500 MBq ^137^Cs radioactive source placed inside a hangar.

The goals of the tests were the following:

- Assess the potential sensitivity of the front end to EMI and to the vibrational (microphonic) noise generated by the engines and propellers of the UAV;
- Assess the capability of the detection system to correctly identify the hotspot of the radioactive field and the nature of the radioactive source.

**Figure 13:**
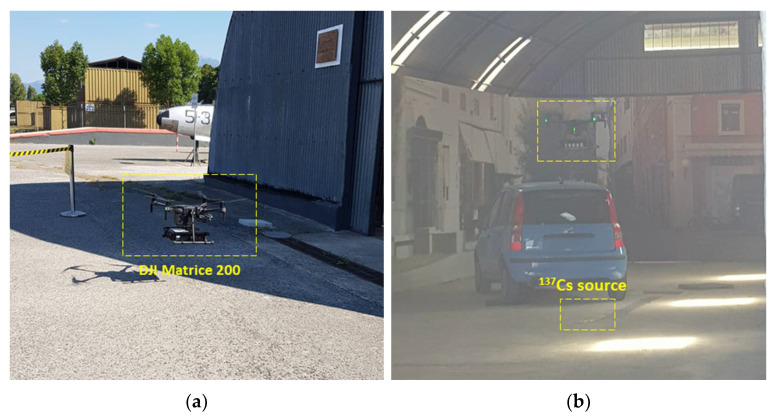
Tests conducted with a UAV mounting the detection units to assess the compatibility of the system with the use in flight. The UAV approaching the radioactive source (**a**); the UAV hovering on top of the radioactive source to assess the activity level and the nature of the emitter (**b**).

The UAV was activated and driven toward the location of the source (which was known) according to the following phases: approaching phase, hoovering and identification phase, and detaching phase (Figure 13).

**Figure 14:**
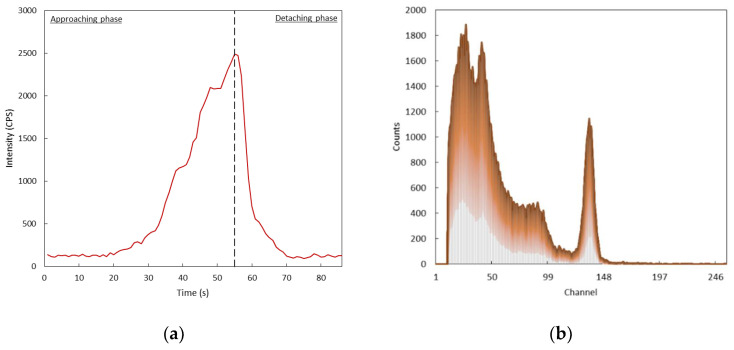
Results of the in-flight testing of the detection system showing the curve corresponding to the detected activity during the (**a**) and the collected spectrum during the flight (**b**).

The activation of the UAV did not produce any difference in the background activity detected by the scintillators, therefore we assumed that the effect of vibrations did not introduce any additional electronic noise to the signals’ path; moreover, radio frequency communication (and any other potential EM noise source) did not affect the response of the system. The system was able not only to identify the hotspot of the radioactive field, but it was also able to identify the nature of the gamma emitter in a few tens of seconds (Figure 14).

The UAV-mounted detection system was also successfully used during a multidisciplinary exercise, where the detecting capabilities of the UAV were tested in a simulation of an emergency RN scenario hold at CEA, Saclay (France).

**Figure 15:**
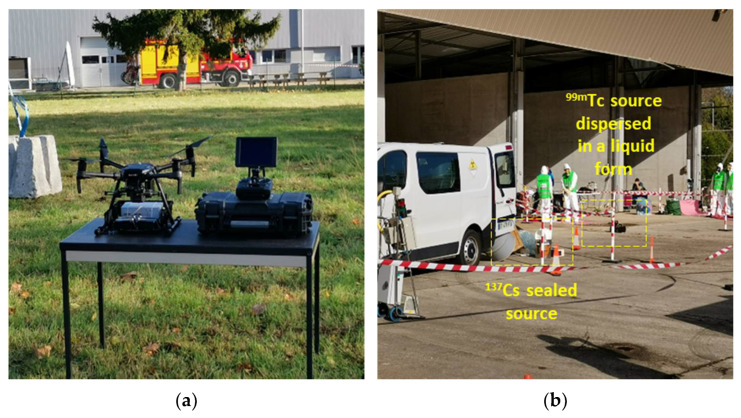
Detection system mounted under the UAV presented at CEA (**a**); radioactive contamination scenario (**b**).

The event was devoted to the identification, recovery, and decontamination from ^137^Cs and ^99m^Tc orphan sources. In such a scenario, the UAV, equipped with the scintillation-based RN detection system, was able to correctly identify the presence of both radioactive sources in a matter of minutes. The ^137^Cs was in the form of a sealed source (showing a very low level of activity), whereas the ^99m^Tc was dispersed in liquid form and diluted for public safety reasons (Figure 15).

**Figure 16:**
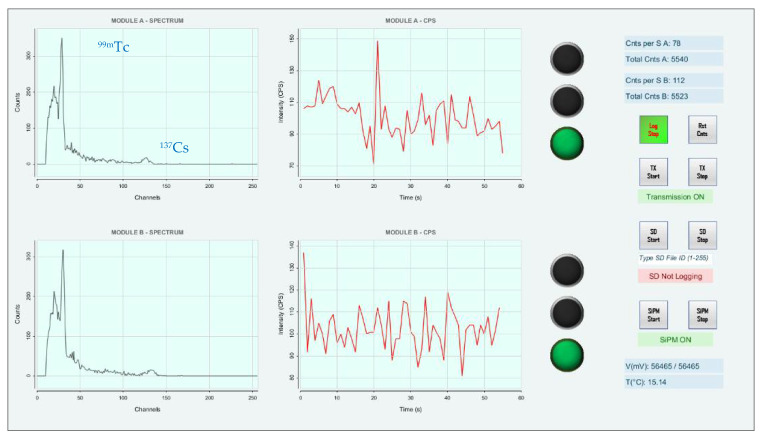
Results of the inspection of the radioactive contamination scenario conducted with the UAV.

The resulting spectra, after only 3 min of hovering phase, are shown in Figure 16. Even without the removal of the background signal, the curves clearly show the presence of the ^137^Cs and ^99m^Tc photopeaks.

To fulfil the tasks of radiation mapping and localization, a pre-built GPS module based around the MTK3339 chipset has already been chosen, but it has not yet been installed. This module is ideal for working on mobile applications such as smartphones, vehicles, personal trackers, digital cameras, and other industrial applications. The high sensitivity (−165 dBm) makes it ideal for dense environments, such as busy cities. It comes with an integrated or external antenna, a built-in data logging feature to record the track, time, and position, and automatic antenna switching. Basically, the MTK3339 is a GPS system on a chip that can monitor up to 22 satellites on 66 channels, and it offers low power operation at only 20 mA during navigation operations. By using a GPS module, another useful feature that may be added to the GUI is a geographic localization framework, which could allow operators to map the measured radioactive activity within a chosen area. With such a module, it will be possible to generate a space-radioactive activity map. By studying the shape of the area to investigate, a measuring path of N points will be drawn; then, by processing the acquired data, the hotspot of the radioactive field will be statistically identified. The framework may be integrated with the QGIS platform. QGIS is a GIS (geographic information system) software, which allows one to analyze and edit spatial data and generate cartography. QGIS supports both vector and raster data as well as major spatial databases such as PostgreSQL/PostGIS or Spatialite. The strength of QGIS is that it integrates the processing algorithms of other open-source projects, such as GRASS GIS and SAGA GIS, in an intuitive interface [42,43].

## 4. Conclusions

In this work, a battery powered, lightweight, compact, and inexpensive gamma-ray detection and spectroscopy system was developed, implemented, and tested. The system is designed to be installed on rotorcraft UAVs with a maximum payload of 25 kg; such UAVs, once equipped with the detection system, may be used to reduce the time and casualties of first responders when managing an emergency involving RN agents.

We first described the phisical principles and the hardware components needed to implement the platform, then we paid attention to the individual stages of the system while recalling some of the concepts behind the technology used. In addition, the actual implementation and the test procedures of the prototype were described and presented along with the results of charge calibration and resolution, recorded spectra, energy calibration, and intrinsic photopeak efficiency. The experiments have demonstrated adequate sensitivity, efficiency, linearity, and noise levels to perform gamma spectroscopy using a CsI(Tl) scintillation detector.

The behaviour of the radiation detection system mounted under a UAV—a DJI Matrice 200—was also tested through measurements conducted in order to assess the potential sensitivity of the unit to EMI and to vibrational noise generated by the engines and propellers of the UAV. Subsequently, the UAV was used to detect and identify the nature of a radioactive contamination in a scenario simulating a RN emergency event.

When developing a new gamma radiation detection system, raw data need to be processed first not only to perform the characterization of the analog front end and of the data acquisition system separately, but also to allow the UAV to carry out the required radiation monitoring tasks. Among them, common tasks are radiation contamination mapping, localization of the radioactive source, and plume-tracking in case of diffused contamination. From a software standpoint, the implementation of such algorithms requires true count and spectroscopy processing of the gamma radiation data and the use of statistical and machine learning methods to process GPS data to allow the drone to track the radioactive plume and to identify the location of the radioactive sources displaced in the area of interest. From an hardware standpoint, the radio communication modules, the memory supports, and the GPS modules chosen are critical to maintain the integrity of the acquired data. The optimization of such aspects shall be the focus of future developments in order to fully industrialize the protoype. The integration of data with a GIS software, which allows one to analyse and edit spatial data and generate cartography, is also a mandatory step toward the finalization of the project.

Summarizing, the aim of the work has been to design, develop, and characterize a scintillation gamma spectroscopy system based mainly on off-the-shelf components and open source software tools. Both the analog front end and the data acquisition system have been developed by minimizing the number of components and the cost of the materials used, making the platform disposable and replaceable without much effort. The detection system was also mounted on board a UAV to assess the compatibility of the mobile platform to be used during in-flight operations. Once equipped with a GPS module integrated with a GIS tool, the platform shall be provided with appropriate algorithms to carry out common radiation monitoring tasks, such as source localization, mapping, and tracking. Finally, such UAV shall be able to perform all the previously noted activities in order to be used by first responders or military personnel during the management of scenarios involving radioactive and nuclear agents to assess the risk when approaching a potentially radiation contaminated area.

## Figures and Tables

**Table 1:** Characteristics of the assembled system.

Parameter	DAQ and RF Unit	Detection Unit
Weight (g)	<400	<200
Size (mm^3^)	220 × 110 × 80	110 × 80 × 38
Power Consumption (mA)	<250	<5
Cost (euro)	<250	<300

**Table 2:** Results of the energy calibration.

E (keV)	Measured Channel	FWHM (Channels)	Energy Resolution (%)	Predicted Channel (Linear Fit)	NL (%)
59	47	8	17.0	49	−4.0
662	465	30	6.5	457	1.7
1173	801	40	5.0	803	−0.2
1332	909	40	4.4	910	−0.2

**Table 3:** Characteristics of the exposures performed to investigate the energy dependence of the intrinsic photopeak efficiency of the detectors.

Emitter	Activity (MBq)	Distance (m)	Exposure Time (min)
^241^Am	5350	5	1
^137^Cs	782	3	10
^60^Co	110	1.7	10

## Data Availability

Open source. Libraries, if necessary, are released under MIT, Apache, and BSD license. Data presented in the article can be requested by directly contacting the authors.
